# Kidney-Draining Lymph Node Fibrosis Following Unilateral Ureteral Obstruction

**DOI:** 10.3389/fimmu.2021.768412

**Published:** 2021-12-27

**Authors:** Xiaofei Li, Jing Zhao, Said Movahedi Naini, Gianmarco Sabiu, Stefan G. Tullius, Su Ryon Shin, Jonathan S. Bromberg, Paolo Fiorina, George C. Tsokos, Reza Abdi, Vivek Kasinath

**Affiliations:** ^1^ Transplantation Research Center, Brigham and Women’s Hospital, Harvard Medical School, Boston, MA, United States; ^2^ Key Laboratory of Combinatorial Biosynthesis and Drug Discovery, Ministry of Education, and Wuhan University School of Pharmaceutical Sciences, Wuhan, China; ^3^ Division of Engineering in Medicine, Department of Medicine, Brigham and Women’s Hospital, Harvard Medical School, Cambridge, MA, United States; ^4^ Departments of Surgery and Microbiology and Immunology, Center for Vascular and Inflammatory Diseases, University of Maryland School of Medicine, Baltimore, MA, United States; ^5^ Division of Rheumatology and Department of Immunology, Beth Israel Deaconess Medical Center, Boston, MA, United States

**Keywords:** unilateral ureteral obstruction (UUO), lymph node, fibroblastic reticular cell (FRC), renal immune homeostasis, kidney fibrosis, chronic kidney disease, acute kidney injury

## Abstract

Although the primary organ has been the subject of intense investigation in the field of organ fibrosis over the past several decades, the presence of lymph node fibrosis due to persistent activation of the immune response in its partner organ remains largely unknown. Previously, we demonstrated that activation of the immune response following ischemia-reperfusion injury (IRI) and crescentic glomerulonephritis (CGN) in the kidney was associated with extracellular matrix (ECM) production by fibroblastic reticular cells (FRCs) of the kidney-draining lymph node (KLN). Here, we sought to determine whether FRCs in the KLN become similarly fibrogenic following unilateral ureteral obstruction (UUO) of the kidney. We subjected 6–8-week-old C57BL/6J mice to UUO for 2, 7, and 14 days. We examined the microarchitecture of the kidney and KLN by immunofluorescence staining at each timepoint, and we quantified immune cell populations in the KLN by flow cytometry. The contralateral kidney unaffected by UUO and its partner KLN were used as controls. We found through immunofluorescence staining that FRCs increased production of ECM fibers and remodeled the microarchitecture of the UUO KLN, contributing to fibrosis that mirrored the changes in the kidney. We also observed by flow cytometry that the populations of CD11b^+^ antigen-presenting cells, CD11c^+^ dendritic cells, and activated CD4^+^ and CD8^+^ T cells were significantly higher in the UUO KLN than the KLN draining the unaffected contralateral kidney. Expression of the TGFβ/TGFβR signaling pathway was upregulated and colocalized with FRCs in the UUO KLNs, suggesting a possible mechanism behind the fibrosis. Both release of ureteral ligation at 2 days following UUO and depletion of FRCs at the time of injury onset halted the progression of fibrosis in both the kidney and the KLN. These findings for the first time highlight the association between fibrosis both in the kidney and the KLN during UUO, and they lay the groundwork for future studies that will investigate more deeply the mechanisms behind the connection between FRCs and KLN fibrosis.

## Introduction

Organ fibrosis has been the subject of intense research over the decades, but the development of fibrosis in draining lymph nodes (DLNs) due to the inflammation and injury responses in the upstream tissues remains unknown.

Lymph nodes (LNs) are the primary sites where the adaptive immune response is determined following injury to the corresponding organ, and increasing evidence indicates that the stromal compartment of DLNs is essential to striking a balance between immunity and tolerance (1). Fibroblastic reticular cells (FRCs) are the major cells that populate the stromal compartment of the T cell zone in the DLN, and they perform several essential functions that maintain the structural and immunological homeostasis of DLNs, including the production of extracellular matrix (ECM) fibers (2). We have demonstrated previously the critical importance of the activity of FRCs in the kidney-draining lymph node (KLN) to the immune response in mouse models of renal ischemia-reperfusion injury (IRI) and crescentic glomerulonephritis (CGN) (3, 4). FRCs inhibit pro-inflammatory properties of T cells through direct contact (1). The loss of the tolerogenic effects of this contact, due to separation of T cells from FRC by the buildup of ECM fibers, may lead to an unchecked local adaptive immune response that results in organ fibrosis. In the IRI study, we found that fibrotic remodeling of the KLN correlated with a higher severity of renal damage following recurrent IRIs (4). However, the importance of FRCs and deposition of ECM in the stromal compartment of the KLN to kidney damage during unilateral ureteral obstruction (UUO) have not been studied before.

UUO is a common preclinical model of chronic kidney disease, and its correlate of obstructive nephropathy in human patients is an important cause of renal damage that occurs commonly following compression of the ureter, often by a malignant intraabdominal or intrapelvic lesion (5). Major advantages to UUO as an *in vivo* model of renal damage include its role as a model of both acute and subacute kidney injury as well as the presence of the contralateral kidney in the animal as a negative control. Numerous previous studies have detailed the molecular pathways that cause fibrosis of the kidney during UUO, and blockade or ablation of these signaling cascades holds major clinical significance for potential prevention of chronic kidney disease (6, 7). Although the presence of a single functional kidney in UUO precludes the usefulness of serological markers, such as blood urea nitrogen (BUN) and serum creatinine (Cr), for assessment of renal damage, the accumulation of fibrosis in the obstructed kidney correlates with its loss of function over time (7). This fibrosis in the obstructed kidney during UUO has been investigated extensively across many years and by a multitude of investigators, but nobody has found whether similar changes occur simultaneously in the stromal compartment of the KLN.

In this study, we performed UUO in mice and examined the correlation between fibrosis in the kidney and ECM deposition by FRCs in the KLNs up to 14 days following ureteral ligation. We found that the density of ECM fibers increased in the UUO KLNs over time, and that FRCs in the KLN produced these fibers. Early release of ureteral ligation 2 days following UUO halted the progression of fibrosis in both the kidneys and KLNs. Ablation of FRCs also resulted in less extensive fibrosis in both the kidney and KLN. These observations reveal new mechanisms of LN fibrosis and set the stage for investigating the utility of entirely innovative therapies targeting FRCs as a novel adjunct treatment for organ fibrosis, for which no therapy exists currently.

## Materials and Methods

### Mice

6-8-week-old C57BL/6J mice (Jackson Laboratory, Bar Harbor, ME) were used in all experiments. UUO was performed by ligation of the left ureter as described (7), and the mice were euthanized 2, 7, or 14 days following the operation. Kidneys and KLNs were harvested at the time of euthanasia and stored at appropriate temperatures for subsequent analysis.

### UUO Release

The UUO release (R-UUO) model was achieved as described by Cochrane et al. (8). Ureteral ligation was performed on the left kidney, and the clamp was removed under general anesthesia 2 days or 7 days after the UUO surgery. The UUO release mice were euthanized at 21 days following UUO. Animal studies were approved and conducted according to the Institutional Animal Care and Use Committee of Brigham and Women’s Hospital, Boston, MA.

### FRC Depletion Using DT

CCL19-Cre (CCL19Cre [Tg (CCL19-cre)489Biat] mice were originally a gift from Shannon Turley (Genentech, South San Francisco, CA). C57BL/6-Gt (ROSA)26Sortm1 (HBEGF)Awai/J (C57BL/6-iDTR) mice were purchased from Jackson Laboratories. The C57BL/6-iDTR mice were backcrossed with the CCL19-Cre mice to create CCL19-Cre x iDTR mice, a transgenic mouse strain of C57BL/6 background in which FRCs can be depleted significantly following administration of diphtheria toxin (DT) (3, 9). These mice were injected with 100 ng of diphtheria toxin (DT) intraperitoneally (i.p.) daily for 5 days following UUO (Days 0-4) to deplete FRCs. CCL19-Cre x iDTR mice that did not receive DT were used as the control group.

### Immunofluorescence Staining

KLNs were frozen and sectioned with a cryomicrotome. After blocking with 3% (vol/vol) bovine serum albumin in phosphate-buffered saline (PBS) for nonspecific antigens, sections were incubated overnight with primary antibodies at 4°C, followed by secondary conjugated antibodies for 1 hour at room temperature. The following antibodies were used: rat anti-MECA79 (Novus Biologicals, 1:200), goat anti-PDPN (R&D Systems, 1:200), rabbit anti-fibronectin (Abcam, 1:300), rabbit anti-collagen I (Abcam,1:300), rabbit anti-LYVE-1 (Abcam, 1:300), fluorescein (FITC)-conjugated rat anti-LYVE-1 (BioLegend, 1:100), and rat anti-TGFβR1 (Santa Cruz, 1:200). The secondary antibodies used were either FITC-anti-rat, Cy3-anti-rabbit/goat, or aminomethylcoumarin acetate (AMCA)-anti-goat (Jackson ImmunoResearch, 1:200). Images were captured using a EvosFL Auto2 fluorescence microscope. Areas of positive staining were assessed semi-quantitatively by ImageJ software (NIH). Statistical analysis was performed using two-tailed Student’s t tests by Prism (GraphPad Software, Inc).

### Real-Time PCR Experiments

Kidneys and KLNs were stored at -80°C after harvest from the mice. RNA from KLNs was isolated by Direct-zol RNA Miniprep Plus kit (Zymo research, Irvine, CA), as per the manufacturer’s protocol. The following primers were used: *Gapdh* forward 5’-AGCCACATCGCTCAGACAC-3’ and reverse 5’-GCCCAATACGACCAAATCC-3’; *Tgfb1* forward 5’-CAACAATTCCTGGCGTTACCTTGG-3’ and reverse 5’-GAAAGCCCTGTATTCCGTCTCCTT-3’; *Tgfbr1* forward 5’-TGCCATAACCGCACTGTCA-3’ and reverse 5’-AATGAAAGGGCGATCTAGTGATG-3’; *Tgfbr2* forward 5’- AGCATCACGGCCATCTGTG-3’ and reverse 5’-TGGCAAACCGTCTCCAGAGT-3’; *Smad7* forward 5’-GGGCTTTCAGATTCCCAACTT-3’ and reverse 5’-CACGCGAGTCTTCTCCTCC-3’; *Bmp7* forward 5’-CAAGCAGCGCAGCCAGAATCG-3’ and reverse 5’-CAATGATCCAGTCCTGCCAGCCAA-3’.

### Flow Cytometry

KLNs were harvested at time of euthanasia, then crushed and filtered through a 70-μm cell strainer. The resulting single-cell suspension was centrifuged at 1,500 rpm for 5 minutes, resuspended in DMEM, then counted manually utilizing 0.4% trypan blue (Thermo Fisher Scientific) to exclude dead cells. The cell suspension was placed in 96-well round-bottom plates for intracellular cytokine staining. The cells were first incubated with phorbol 12-mystirate 13-acetate (100 ng/ml) (Sigma-Aldrich), ionomycin (1 mg/ml) (Sigma-Aldrich), and GolgiStop protein transport inhibitor (BD Biosciences, San Jose, CA) at 37°C for 4 hours. Then the cells were washed and stained according to standard protocols, and flow cytometry was performed *via* BD FACSCanto II flow cytometer (BD Biosciences). Results were analyzed using FlowJo software (FlowJo LLC). All antibodies were purchased from BD (Becton Dickinson Franklin Lakes, NJ).

### Electron Microscopy

KLNs were fixed in Karnovsky fixative and processed as previously described (4). Sections were visualized using an FE-SEM (Zeiss Crossbeam 540), using the aSTEM detector.

### Statistical Analysis

Data are presented as means ± SEM. Differences between two groups were analyzed for significance by unpaired two-tailed Student’s t test, and differences among groups were analyzed for significance by analysis of variance (ANOVA). P values less than 0.05 were considered significant. All statistical analysis was performed using GraphPad Prism.

## Results

### Podoplanin (PDPN)^+^ FRCs Produce ECM Fibers More Extensively in UUO KLNs

**Figure 1:**
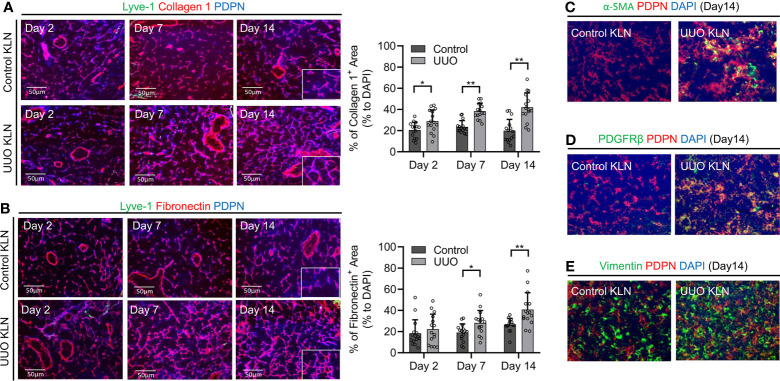
Contribution of FRCs to increase in ECM density in the KLN over time. **(A)** Fluorescence micrographs of Lyve-1^+^ lymphatic vessels (green), collagen 1 fibers (red), and PDPN^+^ cells (blue) demonstrate increased density of collagen I fibers and colocalization with Lyve-1^-^PDPN^+^ FRCs through Day 14. Semiquantitative analysis and comparison of collagen I^+^-stained area between the UUO and contralateral (control) KLNs show significantly higher density of collagen 1^+^ fibers normalized to DAPI-positive area in the UUO KLN at all time points (n=15 random fields of sections from 3 mice). **(B)** Fluorescence micrographs of Lyve-1^-^PDPN^+^ FRCs (blue) and fibronectin fibers (red) show increased density of fibronectin fibers and colocalization with FRCs through Day 14. Semi-quantitative analysis and comparison of fibronectin^+^-stained area shows significantly higher density of fibronectin fibers at Days 7 and 14 in UUO KLN as compared to contralateral (control) KLN (n=15 random microscopic fields of sections from 3 mice). **(C–E)** Fluorescence micrographs of UUO KLN and contralateral (control) KLN sections at Day 14 demonstrate increased expression of **(C)** α-SMA (green), **(D)** PDGFRβ (green), and **(E)** vimentin (green), and increased colocalization (yellow) with PDPN^+^ FRCs (red) in UUO KLN at Day 14 (DAPI nuclear stain, blue). Data represented by means ± SEM. *p < 0.05, **p < 0.01.

As the prominent cells of the stromal compartment in DLNs, FRCs are PDPN^+^ resident stromal cells that support the integrity of the microarchitecture by producing ECM. We have demonstrated previously the importance of ECM deposition and fibrosis in the KLN to the pathogenesis of two other preclinical kidney disease models: renal ischemia-reperfusion injury (4) and glomerulonephritis (3), In those studies, we found that the activity of FRCs is critical to the fibrogenesis in the KLN. However, the contributions of FRCs and ECM deposition in the KLN to the pathogenesis of UUO have not been investigated. Therefore, we examined the KLNs in the UUO model at different time points following ureteral ligation for histological evidence of these changes. To identify the contribution of FRCs in the KLNs after UUO, we investigated whether PDPN colocalized with the ECM markers collagen 1 and fibronectin through immunofluorescence staining. We noticed that the collagen 1 and fibronectin fibers colocalized with PDPN^+^LYVE-1^-^ cells, the molecular signature consistent with FRCs, at 2 days, 7 days, and 14 days (Days 2, 7, and 14) following UUO (**Figures 1A, B**). In addition, the density of collagen fibers was significantly higher in the UUO KLN than the KLN draining the unaffected contralateral kidney at all three time points, and the fibronectin fiber density was significantly higher at Days 7 and 14. Moreover, we found through co-staining of PDPN^+^ cells with αSMA, PDGFRβ, and vimentin that FRCs in the UUO KLNs expressed significantly higher levels of these fibrosis markers than those in the contralateral KLNs (**Figures 1C–E** and **Supplementary Figures 1A–C**). Electron micrographs of the UUO KLN at Day 14 also revealed evidence of extensive ECM deposition within fibrils extending from FRCs (**Supplementary Figure 1D**). This fibrosis was also reflected in a higher overall density of ECM fibers in the UUO KLNs, as indicated by Masson’s trichrome stain (**Supplementary Figure 1E**).

### Antigen-Presenting Cells Infiltrate the KLN, and T Cells Are Activated Within the KLN Following UUO

**Figure 2:**
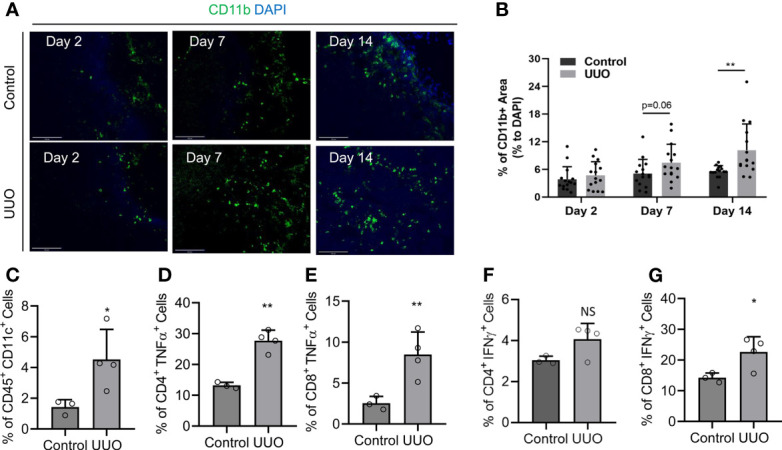
Infiltration of antigen-presenting cells and activation of lymphocytes in the UUO KLN. **(A, B) (A)** Fluorescence micrographs of CD11b^+^ antigen-presenting cells (green) and **(B)** semiquantitative analysis of CD11b^+^ staining normalized to DAPI^+^ area (blue) indicate that the density of CD11b^+^ antigen-presenting cells increases in the UUO KLN over time, and it is significantly higher than the contralateral (control) KLN at Day 14. **(C–G)** Flow cytometric analysis of KLNs at Day 14 indicate significantly higher percentages of **(C)** CD45^+^CD11c^+^ dendritic cells, **(D)** CD4^+^TNFα^+^ T cells, **(E)** CD8+TNFα+ T cells, **(F)** CD8^+^IFNγ^+^ T cells, and higher percentage of **(G)** CD4^+^IFNγ^+^ T cells in the UUO KLN than the contralateral (control) KLN at Day 14 (n=3-4 mice). Data represented by means ± SEM. *p < 0.05, **p < 0.01. NS, not significant.

The position of the KLN as the primary secondary lymphoid organ that drains the kidney situates it as the prime site for modulation of adaptive immunity in the kidney, through regulation of the interplay between the innate and adaptive immune responses. Therefore, we sought to investigate this interaction by determining whether macrophage infiltration and T cell activation increase concurrently in the KLN following UUO. We stained the KLNs for CD11b^+^ antigen-presenting cells by immunofluorescence, and we found that they increased progressively through Day 14 (**Figure 2A**). Semiquantitative analysis indicated that the CD11b^+^ macrophage population was higher in the UUO KLN than the contralateral KLN; although the comparison did not quite reach significance (p=0.06) at Day 7, a significant increase (p<0.01) was observed at Day 14 (**Figure 2B**). In addition, the CD11c^+^ dendritic cell population was significantly higher in the UUO KLN, as quantified by flow cytometry (**Figure 2C**). Moreover, flow cytometric analysis revealed that the TNFα-producing CD4^+^ and CD8^+^ T cell populations and IFNγ-producing CD8^+^ T cell population were significantly higher in the UUO KLN (**Figures 2D–F**). The UUO KLN also contained a higher population of CD4^+^IFNγ^+^ cells, although this comparison did not achieve significance (**Figures 2F, G** **)**. Finally, we stained the KLNs for LYVE1^+^ lymphatics, through which antigen-presenting cells likely traffic from the kidney to the KLN (10, 11), and MECA79^+^ high endothelial venules, through which naïve T cells enter the KLN from the systemic circulation. We found that the expansion of LYVE1^+^ lymphatic vessels peaks at Day 14, when the lymphatic vasculature is significantly more extensive than in the contralateral KLN (**Supplementary Figures 2A, B**).

### TGFβ/TGFβR1 Pathway Is Activated in the KLN Following UUO

**Figure 3:**
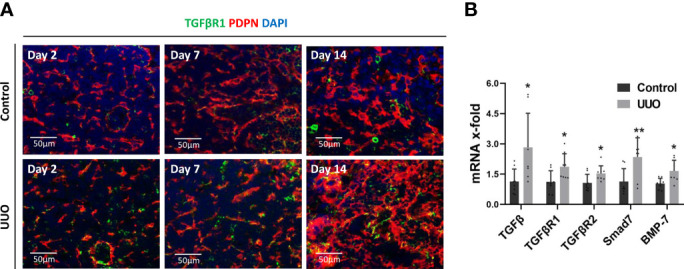
TGFβ/TGFβR signaling pathway is active in the UUO KLN. **(A)** Fluorescence micrographs of UUO and contralateral (control) KLNs demonstrate co-staining (yellow) of TGFβR1 (green) with PDPN^+^ FRCs (red) through Day 14. **(B)** RT-PCR shows significantly higher expression of TGFβ, TGFβR1, TGFβR2, Smad7, and BMP-7 in UUO KLN than contralateral KLN at Day 7. Each sample was performed in duplication (n=3). Data represented by means ± SEM. *p < 0.05, **p < 0.01.

Expression of TGFβ has been linked to fibrosis in secondary lymphoid organs in preclinical HIV models, suggesting a possible signaling pathway that could be responsible for the fibrosis observed in the KLN (12). Hence, we interrogated whether the TGFβ/TGFβR1 pathway is activated in the KLN following UUO. We found through co-staining of PDPN^+^LYVE-1^-^ cells with TGFβR1 that FRCs in the UUO KLNs expressed higher amounts of TGFβR1 than those of contralateral KLNs (**Figure 3A**), indicating a possible route for the increase in fibrosis. Gene expression levels of TGFβ, TGFβR1, TGFβR2, the GFβ regulator Smad7, and the TGFβ family member BMP-7 were all significantly higher in the UUO KLNs at Day 7 as compared to the contralateral KLNs (**Figure 3B**). These data suggest that the TGFβ signaling pathway could function as a chief mediator of ECM production by FRCs in the KLN during UUO.

### Release of Ligation at 2 Days Following UUO Halts Kidney and KLN Fibrosis but Does Not Alter the Course at 7 Days

**Figure 4:**
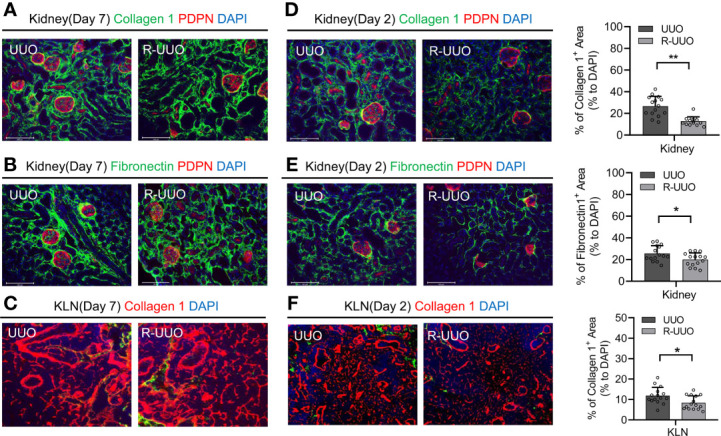
Release of UUO (R-UUO) at Day 2 halts fibrosis in the kidney and KLN but has no effect at Day 7. **(A, B)** Fluorescence micrographs of collagen 1 fibers (green) and fibronectin fibers (green) reveal no difference in fibrosis between the UUO and R-UUO kidneys at Day 21 following release of ureteral ligation at Day 7 (PDPN glomerular stain, red; DAPI nuclear stain, blue). **(C)** Fluorescence micrographs of collagen 1 fibers (red) demonstrate no difference in fibrosis between the UUO and R-UUO KLNs at Day 21 following release of ureteral ligation at Day 7 (DAPI nuclear stain, blue). **(D, E)** Fluorescence micrographs and semiquantitative analysis show significantly higher density of **(D)** collagen 1 fibers (green) and **(E)** fibronectin fibers (green) normalized to DAPI-stained area (blue) in UUO kidneys than R-UUO kidneys at Day 21 following release of ureteral ligation at Day 2 (PDPN glomerular stain, red) (n=15 random microscopic fields of sections from 3 mice). **(F)** Fluorescence micrographs and semiquantitative analysis show significantly higher density of collagen 1 fibers (green) normalized to DAPI-stained area (blue) in UUO KLNs than R-UUO KLNs at Day 21 following release of ureteral ligation at Day 2 (n=15 random microscopic fields of sections from 3 mice). Data represented by means ± SEM. *p < 0.05, **p < 0.01.

A major question within the field of chronic kidney disease is whether the fibrosis associated with impaired kidney function can be reversed. Previous studies of UUO mouse models have sought to answer this question by releasing the ligated ureter at various time points following UUO. These experiments have demonstrated mixed results— the fibrosis that occurs in the kidney following UUO may or may not reverse after the obstruction has resolved (8, 13, 14). However, whether the fibrosis in the KLN resolves following release of ureteral ligation has never been published. Here, we released the ureter from ligation at Day 7 and euthanized the mice at Day 21. Then, we performed immunofluorescence staining to compare the fibrosis in both the kidney and the KLN to the corresponding organs of the mice in which the ureter remained ligated for 21 days. First, we found no significant difference in the extent of fibrosis between the two kidneys, as shown by both collagen I and fibronectin (**Figures 4A, B**). Then, we sought to determine whether this persistence in fibrosis is reflected in the KLN, and we found by immunofluorescence staining that the density of ECM fibers in the KLN was also similar between the UUO and R-UUO groups (**Figure 4C**). Then, we investigated whether releasing the ureteral ligation at an earlier time point could halt progression of fibrosis by releasing the ureters at Day 2 and euthanizing the mice at Day 21. The fibrosis in the R-UUO KLN, as determined by collagen 1 and fibronectin staining, was significantly less dense than the UUO KLN (**Figures 4D, E**). Semiquantitative analysis indicated that the density of collagen I fibers in the R-UUO KLN also was significantly lower, as compared to the UUO KLN (**Figure 4F**).

### Depletion of FRCs Is Associated With Less Extensive Fibrosis in the Kidney and KLN Following UUO

**Figure 5:**
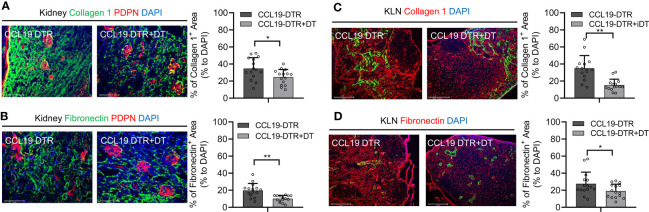
Depletion of FRCs reduces fibrosis in KLNs and kidneys following UUO. **(A, B)** Fluorescence micrographs and semiquantitative analysis reveal significantly lower density of **(A)** collagen 1 fibers (green) and **(B)** fibronectin fibers (green) normalized to DAPI-stained area (blue) in KLNs from CCL19-Cre x iDTR mice following FRC depletion (+DT) at Day 14 (n=5 random microscopic fields of sections from 3 mice). **(C, D)** Fluorescence micrographs and semiquantitative analysis reveal significantly lower density of **(C)** collagen 1 fibers (green) and **(D)** fibronectin fibers (green) normalized to DAPI-stained area (blue) in kidneys from CCL19-Cre x iDTR mice following FRC depletion (+DT) at Day 14 (PDPN glomerular stain, red) (n=15 random microscopic fields of sections from 3 mice). Data represented by means ± SEM. *p < 0.05, **p < 0.01.

As demonstrated here in **Figure 1**, FRCs colocalize closely with the nidus of fibrogenesis that appears in the KLN following UUO. Therefore, we investigated whether FRCs are the major source of fibrosis in the UUO KLN, through the use of CCL19-Cre x Rosa26-diphtheria toxin receptor (CCL19-Cre x iDTR) mice, a transgenic mouse strain of C57BL/6 background in which FRCs can be depleted significantly following administration of diphtheria toxin (DT) (3, 9). We demonstrated in previous studies of renal IRI and CGN in mouse models that depletion of FRCs was associated with lower kidney damage (3, 4). Here, we sought to determine whether depletion of FRCs would exert similar amelioration of kidney injury following UUO. In this model, we injected C57BL/6 mice with 100 ng of DT daily on 5 days following UUO and euthanized the mice at Day 14. Semiquantitative analysis of immunofluorescence staining of the KLNs revealed significantly lower density of collagen I (**Figure 5A**) and fibronectin fibers (**Figure 5B**) following depletion of FRCs in comparison to the CCL19-Cre x iDTR mice that did not receive DT. Interestingly, this reduction in fibrosis in the KLN was accompanied by a similar significant decrease in fibrosis in the kidneys of the mice that underwent FRC depletion, as reflected by immunofluorescence staining of collagen I and fibronectin (**Figures 5C, D**). CCL19^+^ cells were non-existent in the control (contralateral) kidney, and they were present in sparse amounts in the UUO kidney. However, these few CCL19^+^ cells did not co-stain with PDPN (**Supplementary Figure 3**). These observations reflect our earlier findings in models of renal IRI and CGN (3, 4), and they reinforce the concept that the FRCs in the KLN function as prominent arbiters of the direction of the immune response and fibrogenesis observed in the kidney following renal injury.

## Discussion

Organ fibrosis is a major subject of investigation, but little is known about fibrosis of the corresponding DLN. Similarly, the importance of LNs to the generation of adaptive immunity is well-established, but the impact on the immune response of massive, continuous changes to the stromal compartment of a DLN during chronic organ dysfunction represents a less explored area of research.

DLNs receive lymphatic drainage from their corresponding organs *via* afferent lymphatic vessels. This anatomical position situates DLNs as prime surveillance sites for the immunological milieu in the organs from which they collect the lymph. Moreover, millions of naive immune cells enter LNs *via* HEVs daily. Therefore, DLNs are dynamic organs wherein antigens are shuttled continuously *via* lymphatics by dendritic cells that present them to T cells that arrive *via* HEVs (15, 16).

FRCs are the prominent cells of the paracortical stroma in DLNs, and they have a well-characterized dual phenotype, typified by both pro-inflammatory and immunoregulatory properties (17). These multifaceted aspects of FRCs are apparent during the immune response to organ injury, as they proliferate and produce ECM during the early phase, expanding the size of the LN and permitting space for the influx of lymphocytes and antigen-presenting cells (18). However, during the late phase, these pro-inflammatory properties of FRCs must resolve, or the FRCs must adopt anti-inflammatory properties to permit the LN to return to its steady state and prevent prolonged inflammation, which can result in fibrosis of the LN (18). Interestingly, Gregory et al. have shown that an expanded FRC network with quiescent gene expression in the LN is associated with the resolution of a viral infection, and this “primes” the LN to respond to future infections (19). On the other hand, dysregulation of the stromal compartment of the LN results in fibrosis, as has been described in studies of patients infected with human immunodeficiency virus (HIV) (20). Therefore, perturbation of this fragile balance in between healing and fibrosis in the LN that leads to hypersecretion of ECM fibers by FRCs is one possible mechanism behind the fibrosis we have observed in the KLN following recurrent or continuous kidney injuries in the IRI and UUO models, respectively.

The interactions of FRCs with T cells are crucial particularly to guiding the direction of adaptive immune responses that contribute to organ damage following an immunogenic or non-immunogenic insult (21). Though we have demonstrated the importance of FRCs in the KLNs to the pathogenesis of a classically cell-mediated disease, such as CGN (3), we have also shown that the activity of FRCs is equally critical to kidney damage in an insult of primarily vascular origin, such as IRI (4). In the latter investigation, we demonstrated that fibrosis within the kidney caused by repetitive IRI was associated with fibrosis and dysregulation of the stromal compartment of the KLN (4). This fibrosis may have interfered with anti-inflammatory effects of FRCs, for which contact with T cells are requisite.

In this study, we sought to determine whether the microarchitecture of the KLN was subject to similar changes as the kidney became fibrotic during UUO, and whether FRCs were implicated in these changes. We found that production of collagen 1 and fibronectin fibers by FRCs led to dramatic remodeling of the KLN stroma during UUO. Interestingly, we found that both fibers increased in the UUO KLN over time, and this increase occurred in parallel to the expansion of the lymphatic vasculature.

LN fibrosis has been described previously in preclinical models of human/simian immunodeficiency virus (HIV/SIV) infection, and pronounced scarring is seen at late stages (12, 22). This scarring has been attributed to sequelae from chronic activation of the immune response (12). However, LN fibrosis has not been studied extensively in other models. Here, we establish for the first time that the KLN undergoes frank fibrosis during UUO. The data from this study in combination with the findings from our previous investigations of renal IRI and CGN suggest that FRC activation in the KLN is a common correlate of the immune activation caused by kidney injury, irrespective of its primary origin—immunogenic or non-immunogenic. Thus, the process of FRC activation and ECM deposition in the KLN appears to be a common pathway following immune-mediated, ischemic, and mechanical sources of kidney injury. However, the specific mechanisms by which fibrosis in the KLN impact immunity and kidney damage remain unclear.

CD11c^+^ dendritic cells have been found previously to circulate between the kidney and the KLN constitutively in usual states of health (10, 23) and to migrate to the KLN following immune challenge with ovalbumin in mice (11). Here, we demonstrate similarly that the population of CD11b^+^ and CD11c^+^ antigen-presenting cells in the KLN increases following UUO. This increase is accompanied by a higher population of activated CD4^+^ and CD8^+^ T cells in the UUO KLN, a finding that reflects previous data following ovalbumin administration, IRI, and CGN (3, 4, 11). These expanding T cell subsets could migrate subsequently to the kidney. In future studies, we could investigate whether these CD4^+^ and CD8^+^ T cells and their descendants home to the kidney during UUO following adoptive transfer of fluorescently labeled naïve T cells in lymphocyte-deficient RAG-1^-/-^ mice.

Organ fibrosis is thought to be an irreversible condition, during which organ function is lost irretrievably in part due to dysregulation of the stromal compartment. In contrast, the stromal compartment of the LN has the capacity to regenerate following extensive scarring. This capability may arise from the fact that leukocytes vastly outnumber the stromal cells in the LN, which constitute merely ~1% of the cellular population (24). Furthermore, the beneficial effects of preventing the remodeling of the KLN stroma may be suggested by our finding that administering unstimulated FRCs to mice undergoing recurrent renal IRI halted fibrotic changes in the KLN and blunted renal damage (4). Therefore, future investigations could test the efficacy of FRC therapy as a method of prevention for organ fibrosis. Whether administering FRCs will reverse fibrosis in the DLN is another important question that also must be addressed.

The balance between expression of the pro-inflammatory, fibrogenic phenotype and immunoregulatory phenotype in FRCs of the UUO KLN could be crucial to the pathogenesis of kidney injury in this model. TGFβ is known as an important cytokine involved in organ scarring and fibrosis, and our study suggests that activation of the TGFβ-TGFβR signaling pathway should be examined more closely in FRCs of the UUO KLN for its effect on their expression of ECM genes. The source of the TGFβ gene expression in the UUO KLN is unclear, although the antigen-presenting cells that arrive from the kidney are potential culprits. Future research is needed to identify the signaling pathways that govern the important transition of FRCs to an immunoregulatory phenotype, as these pathways are not well understood currently. The lymphotoxin-β receptor (LTβR) signaling pathway is one possible mechanism responsible for control of this phenotypic switch, as previous studies have highlighted its importance in guiding the activity of FRCs during an immune response (1). Thus, blockade of signals that govern formation of the pro-inflammatory phenotype of FRCs and enhancement of those that potentiate anti-inflammatory their properties could serve to reduce kidney damage following UUO.

## Data Availability Statement

The raw data supporting the conclusions of this article will be made available by the authors, without undue reservation.

## Ethics Statement

The animal study was reviewed and approved by the Institutional Animal Care and Use Committee (IACUC) of Brigham and Women’s Hospital.

## Author Contributions

Conceptualization: XL, RA, and VK. Formal analysis: XL, RA, and VK. Investigation: XL and JZ. Resources: RA and VK. Writing – original draft: XL, RA, and VK. Writing – review and editing: XL, SN, GS, ST, SS, JB, PF, GT, RA, and VK. Project administration: XL, JZ, RA, and VK. Supervision: RA and VK. Funding acquisition: RA and VK. All authors contributed to the article and approved the submitted version.

## Funding

This work was supported by the NIH [K08DK124685 (VK), R01AI156084 (RA), and P01AI153003 (RA, JB)] and a grant from Dialysis Clinic, Inc. (VK). The funder was not involved in the study design, collection, analysis, interpretation of data, the writing of this article, or the decision to submit it for publication.

## Conflict of Interest

The authors declare that the research was conducted in the absence of any commercial or financial relationships that could be construed as a potential conflict of interest.

## Publisher’s Note

All claims expressed in this article are solely those of the authors and do not necessarily represent those of their affiliated organizations, or those of the publisher, the editors and the reviewers. Any product that may be evaluated in this article, or claim that may be made by its manufacturer, is not guaranteed or endorsed by the publisher.
