# Supplementary figures and images for: Kidney-Draining Lymph Node Fibrosis Following Unilateral Ureteral Obstruction
Source: Front Immunol. 2021 Dec 27;12:768412. doi: 10.3389/fimmu.2021.768412 (PMC8744208; doi:10.3389/fimmu.2021.768412)

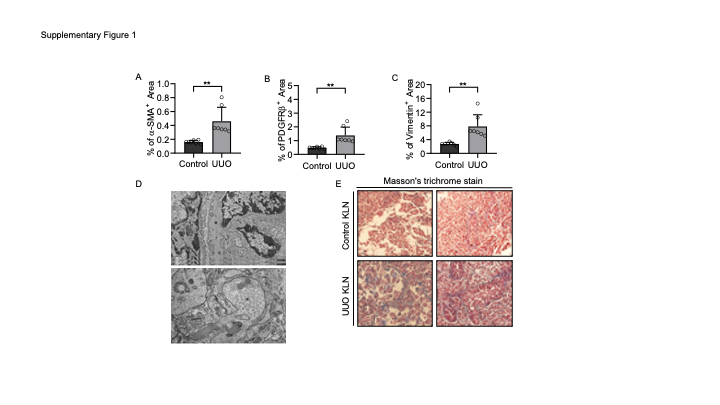

Supplement: Supplementary file 1 [file Image_1.tiff]

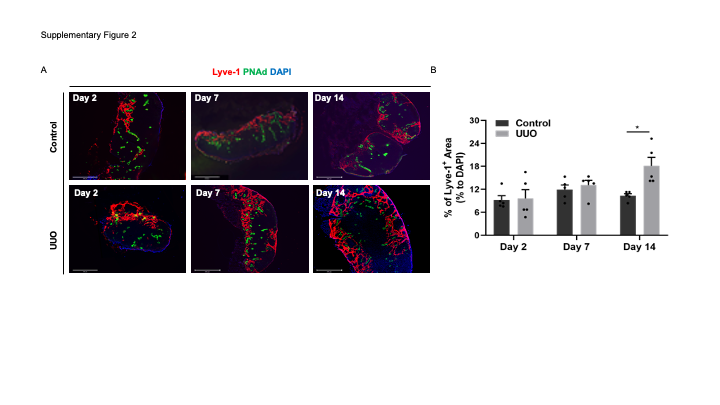

Supplement: Supplementary file 2 [file Image_2.tiff]

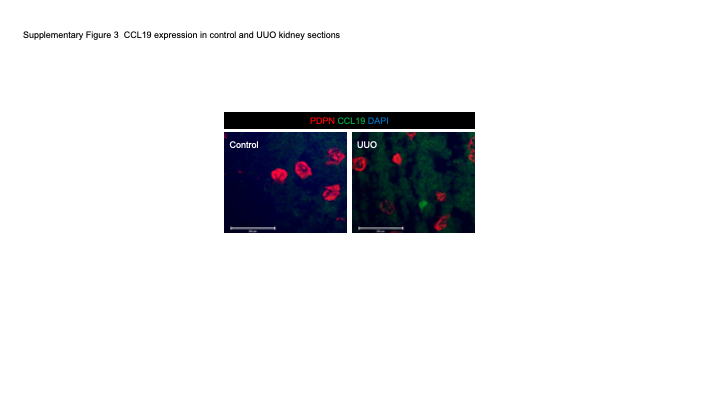

Supplement: Supplementary file 3 [file Image_3.tiff]
